# Serial small- and wide-angle X-ray scattering with laboratory sources

**DOI:** 10.1107/S2052252522007631

**Published:** 2022-08-13

**Authors:** Mark A. Levenstein, Karen Robertson, Thomas D. Turner, Liam Hunter, Cate O’Brien, Cedrick O’Shaughnessy, Alexander N. Kulak, Pierre Le Magueres, Jakub Wojciechowski, Oleksandr O. Mykhaylyk, Nikil Kapur, Fiona C. Meldrum

**Affiliations:** a Université Paris-Saclay, CEA, CNRS, NIMBE, 91191 Gif-sur-Yvette, France; b Department of Chemical and Environmental Engineering, University of Nottingham, University Park, Nottingham NG7 2RD, United Kingdom; c School of Chemistry, University of Leeds, Woodhouse Lane, Leeds LS2 9JT, United Kingdom; d Soft Matter Analytical Laboratory, Department of Chemistry, The University of Sheffield, Brook Hill, Sheffield S3 7HF, United Kingdom; e Rigaku Americas Corporation, 9009 New Tails Drive, The Woodlands, TX 77381, USA; f Rigaku Europe SE, Hugenottenallee 167, Neu-Isenburg 63263, Germany; g School of Mechanical Engineering, University of Leeds, Woodhouse Lane, Leeds LS2 9JT, United Kingdom; University of Iowa, USA

**Keywords:** serial SAXS, serial WAXS, rapid structural analysis, crystallization, microfluidics

## Abstract

Recent advances in X-ray instrumentation and sample injection systems have enabled serial crystallography of protein nanocrystals and the rapid structural analysis of dynamic processes. However, this progress has been restricted to large-scale X-ray free-electron laser (XFEL) and synchrotron facilities, which are often oversubscribed and have long waiting times. Here, we explore the potential of state-of-the-art laboratory X-ray systems to perform comparable analyses when coupled to micro- and millifluidic sample environments. Our results demonstrate that commercial small- and wide-angle X-ray scattering (SAXS/WAXS) instruments and X-ray diffractometers are ready to access samples and timescales (≳5 ms) relevant to many processes in materials science including the preparation of pharmaceuticals, nanoparticles and functional crystalline materials. Tests of different X-ray instruments highlighted the importance of the optical configuration and revealed that serial WAXS/XRD analysis of the investigated samples was only possible with the higher flux of a microfocus setup. We expect that these results will also stimulate similar developments for structural biology.

## Abstract 2

Commercial X-ray scattering instruments were assessed for their ability to perform rapid flow-based structural analysis using micro- and millifluidic sample environments. These studies demonstrate that serial collection of small- and wide-angle X-ray scattering data from small-molecule crystals and nanoparticles is now possible with laboratory instruments.

## 1. Introduction

In serial crystallography (SX) and other serial data collection approaches, measurement snapshots are collected rapidly from many separate analytes (10^1^–10^6^) to produce a merged dataset with excellent statistics, while also minimizing the damage to individual analytes from intense electron and X-ray probes (Neutze *et al.*, 2000 ▸; Spence & Doak, 2004 ▸). Microfluidic and other liquid-handling devices have played an important role in the development of these techniques over the past two decades, first serving as nozzles to inject molecules or crystals into the beam (Chapman *et al.*, 2011 ▸), and subsequently for performing more complex mixing operations to initiate reactions in flow (Schmidt, 2013 ▸). This has facilitated the development of ‘mix-and-inject’ experiments, in which time-resolved studies are performed by controlling the time delay between mixing and analysis (Calvey *et al.*, 2019 ▸). Importantly, time resolution in these experiments is determined by the beam size and the velocity of the flow/analyte as it transits the beam rather than the cumulative acquisition time of the merged dataset (Vakili *et al.*, 2019 ▸). A number of researchers have made use of these techniques to study dynamic processes including the binding and self-assembly of proteins (Beyerlein *et al.*, 2017 ▸; Saldanha *et al.*, 2017 ▸), the triggering of RNA riboswitches (Stagno *et al.*, 2017 ▸; Ramakrishnan *et al.*, 2021 ▸), and the nucleation and growth of crystals and nanoparticles (Levenstein *et al.*, 2019 ▸; Lange *et al.*, 2020 ▸; Radajewski *et al.*, 2021 ▸). However, the X-ray brilliance normally required for such rapid data acquisition strategies can only be found at X-ray free-electron laser (XFEL) and synchrotron facilities, which have limited experimental time. This makes it difficult for researchers to perform measurements on-demand or to guarantee the regular access required for conducting long-term studies.

In parallel with these developments in serial data collection at large-scale facilities, recent years have seen major technical improvements to laboratory X-ray systems. New rotating anode and liquid metal X-ray sources have significantly increased the available flux of commercial X-ray scattering instruments – even approaching the level of second-generation synchrotrons (Skarzynski, 2013 ▸). Low-noise, hybrid photon counting (HPC) detectors first introduced at the synchrotron (Broennimann *et al.*, 2006 ▸) are now supplied as standard on laboratory systems. Additionally, advances in X-ray optics (Yuriy *et al.*, 2015 ▸) and scatterless slits (Taché *et al.*, 2016 ▸) provide microfocused or highly collimated beams with fewer artefacts. Such is the power of these improvements that it was recently demonstrated that X-ray ptychographic imaging is now possible with a laboratory instrument (Batey *et al.*, 2021 ▸).

Inspired by these advances, we performed a series of micro- and millifluidic experiments at three state-of-the-art laboratory X-ray facilities in order to evaluate their potential for time-resolved serial data collection from dynamic samples. Focusing on applications in materials science, we probed a range of samples including dilute inorganic and organic crystal slurries, suspensions of nanoparticles, and a reactive precipitation process. The samples were introduced into the X-ray beam in segmented water-in-oil flows, which were measured by either simultaneous small-angle and wide-angle X-ray scattering (SAXS/WAXS) or X-ray diffraction (XRD) using fast multi-frame exposures. Though there are some examples of previous flow-based X-ray scattering studies in the laboratory, these have been restricted to single-phase flows of crystal slurries (Hammond *et al.*, 2004 ▸; Dharmayat *et al.*, 2008 ▸) or isotropic suspensions of nanoparticles (Polte *et al.*, 2010 ▸; Chen *et al.*, 2015 ▸; Herbst *et al.*, 2019 ▸) that permit long exposure times (≫1 s) and thus, do not require rapid serial acquisition. Our experiments show that laboratory X-ray systems are now indeed ready to perform rapid structural analysis of fast processes (≪1 s) aided by fluidic devices. We also highlight the importance of optimizing both the X-ray optics and the flow channel width to obtain data with a suitable signal-to-noise ratio. To our knowledge, this represents the first report of serial data collection using a commercial laboratory X-ray source.

## 2. Results and discussion

**Figure 1:**
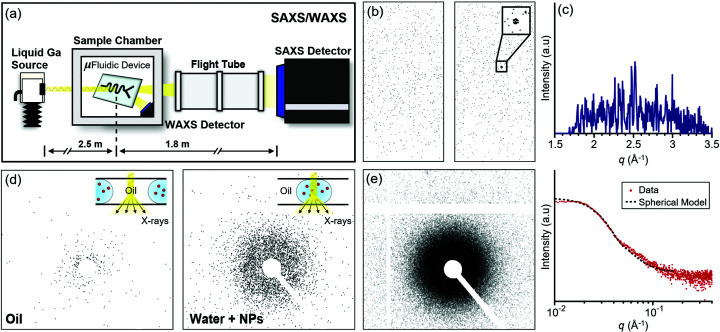
(*a*) Microfluidic experimental setup on the Xeuss 2.0 SAXS/WAXS Laboratory Beamline. (*b*) Representative 2D WAXS patterns from the flow of water-in-oil droplets within the device collected with 0.5 s exposures. The zoomed-in inset shows a possible reflection from the (104) plane of calcite formed during a rapid precipitation process within the droplets. (*c*) Integrated 1D diffraction pattern of the right frame of (*b*) showing that the possible 104 reflection was not preserved during data reduction. (*d*) Representative 2D SAXS patterns collected from 0.5 s exposures that show the characteristic scattering of oil and water droplets containing silica nanoparticles. The insets illustrate the path of the X-ray beam through the microchannel, either encountering the continuous oil phase or a droplet containing nanoparticles. (*e*) Serial 2D (left) and 1D (right) SAXS patterns of silica nanoparticles obtained from automated combination of multi-frame scattering data (30 s cumulative exposure time). The dotted line is a polydisperse sphere form factor fitted to the *I*(*q*) curve taking into account beam convolution.

SAXS/WAXS experiments were performed at the Soft Matter Analytical Laboratory (SMALL) of The University of Sheffield, which is equipped with a Xeuss 2.0 Laboratory Beamline [Xenocs, FR; Figs. 1 ▸(*a*) and S1 of the supporting information]. The Xeuss system at SMALL comprises a high-flux, liquid gallium X-ray source (MetalJet D2+, Excillum, SE) and two HPC detectors: a Pilatus3 R 1M dedicated to SAXS and a customized SWAXS module based on a Pilatus3 R 100k dedicated to WAXS (both Dectris, CH). The X-ray beam is collimated by a three-dimensional single-reflection multi-layered mirror (FOX3D-Ga, Xenocs, FR) combined with two sets of scatterless slits forming a two-pinhole collimator. This setup delivers an almost parallel beam that minimizes smearing of the scattering intensity at small angles. Samples were introduced to the beam using a polymer insert-based microfluidic device comprising two 75 µm-thick polyimide (Kapton) windows for X-ray transmission (Fig. S2). Previously, this device was successfully utilized at three synchrotron beamlines (Levenstein *et al.*, 2019 ▸; Levenstein, Kim *et al.*, 2020 ▸). Aqueous droplets containing the sample materials were formed in FC-40 fluorinated oil at a rate of 1–2 Hz and directed along a 300 × 300 µm flow channel for X-ray analysis with a beam spot size ≃ 250 × 250 µm. Based on the beam size and the droplet velocities used, the dwell time of a particle in the beam was ∼42–96 ms; this equates to the maximum achievable time resolution, neglecting mixing time. The microfluidic device was mounted on a motorized stage within the sample chamber, and X-rays scattered from the device at low angles were carried along an evacuated flight tube to minimize air scattering [Fig. 1 ▸(*a*)]. SAXS/WAXS data are presented as a function of the scattering vector **q** = 4πsinθ/λ, where θ is half the scattering angle (2θ) and λ is the wavelength of the incident X-rays.

For the first test of the SAXS/WAXS system, we studied the rapid precipitation of CaCO_3_ from aqueous solution. Calcite crystals were formed within seconds in the presence of a porous nucleating agent (see the supporting information for experimental details), as confirmed previously by intense X-ray diffraction using a synchrotron source (Levenstein *et al.*, 2019 ▸). In order to capture the dynamics of the flow and to isolate scattering events from the dispersed and continuous phases (Saldanha *et al.*, 2017 ▸), data were initially acquired using consecutive multi-frame exposures of <100 ms per frame. However, almost zero scattering intensity was observed in both SAXS and WAXS with these short exposure times (data not shown). Very few photons were collected in each frame, and separate oil and water scattering could not be distinguished in either WAXS or SAXS even when 0.5 and 1 s exposures at the Nyquist limit of signal acquisition were utilized [Figs. 1 ▸(*b*) and S3] (Levenstein, Kim *et al.*, 2020 ▸). Some 2D features that appeared to correspond to the 104 reflection of calcite were observed, but these were at the same intensity as the experimental noise and were lost upon 1D data reduction [Figs. 1 ▸(*b*) and 1 ▸(*c*)]. These poor scattering statistics also meant that it was impossible to obtain a suitable reference pattern for performing background subtraction.

Next, we studied flowing droplets containing 10 wt% silica (SiO_2_) nanoparticles that had been used previously as an SAXS reference material [Bindzil coloidal silica CC401, AkzoNobel (Mable *et al.*, 2017 ▸)]. Consecutive WAXS patterns remained indistinguishable from one another, whereas SAXS frames corresponding to the oil or to droplets containing nanoparticles could be distinguished with exposures as short as 0.5 s [Fig. 1 ▸(*d*)]. In this case, it was possible to isolate and combine the short SAXS frames from the droplets to produce a single scattering pattern using a serial data processing approach [Fig. 1 ▸(*e*), see the supporting information for experimental details]. This final serial scattering pattern was of sufficient quality to fit with a polydisperse spherical model (Taché *et al.*, 2013 ▸) and attain a nanoparticle diameter of 17.3 ± 4.5 nm, which is within 94% of the value obtained by synchrotron scattering (Mable *et al.*, 2017 ▸). Thus, although the photon flux of the collimated X-ray beam provided by the Xeuss 2.0 was insufficient for extracting a WAXS signal from the crystalline sample studied, satisfactory SAXS data could be obtained with consecutive <1 s exposures from a strongly scattering nanometric sample.

**Figure 2:**
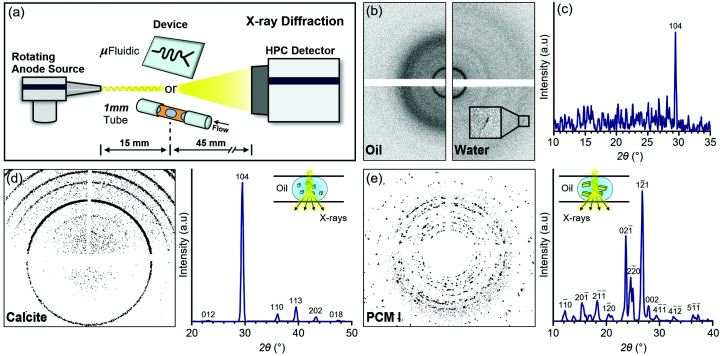
(*a*) Experimental setup on the XtaLAB Synergy-R single-crystal diffractometer illustrating both the microfluidic and the millifluidic devices that were utilized. (*b*) Representative 2D XRD patterns showing the characteristic scattering of water-in-oil droplets within the microfluidic device collected with 25 ms exposures. The zoomed-in inset shows a 104 reflection from calcite. (*c*) Integrated and background-subtracted 1D diffraction pattern of the right frame of (*b*) showing that the 104 reflection was preserved during data reduction. (*d*) Serial 2D (left) and 1D (right) diffraction patterns of calcite crystals obtained from automated background subtraction and combination of multi-frame diffraction data (90 s cumulative exposure time). The diffraction peaks are labelled with their corresponding reflections. (*e*) Serial 2D (left) and 1D (right) diffraction patterns of PCM I crystals obtained from automated background subtraction and combination of multi-frame diffraction data (90 s cumulative exposure time). The diffraction peaks are labelled with their corresponding reflections. The insets in (*d*) and (*e*) illustrate the path of the X-ray beam through the microchannel, where only diffraction frames corresponding to slurry plugs are integrated into the final serial diffraction patterns.

X-ray diffraction (XRD) experiments were performed using an XtaLAB Synergy-R single-crystal diffractometer (Rigaku Oxford Diffraction, PL) comprising a microfocus rotating anode X-ray source (Cu *K*α, PhotonJet-R) and an HPC detector [HyPix-6000HE; Fig. 2 ▸(*a*)] (Le Magueres *et al.*, 2019 ▸). The X-ray beam of the Synergy-R is focused using a pair of multilayer confocal mirrors to maximize the flux at the sample. Since data are only collected at wide angles, it is not essential to minimize beam divergence as with the Xeuss 2.0 SAXS/WAXS instrument. The experiments were performed at either the Rigaku Oxford Diffraction Application Centre (Wrocław, PL) or the Flow-Xl: National Facility for Analysis of Crystallization in Flow Systems, which is currently being commissioned at the University of Leeds (Fig. S1). XRD data are presented as a function of 2θ, by convention the angle between the scattered and the incident beams.

The same insert-based microfluidic platform described above was mounted on a translational stage (XtalCheck-S), and water-in-oil droplets in which calcite crystals were precipitating rapidly were introduced into the X-ray beam (spot size ≃ 140 × 140 µm). Data were acquired with consecutive frames of 25 ms exposure time, and unlike in the previous experiments with the Xeuss 2.0, frames displaying the characteristic scattering of either water or fluorinated oil were readily distinguished [Fig. 2 ▸(*b*)]. Based on the beam size and the droplet velocity, our maximum time resolution was ∼24 ms, very closely matching the chosen exposure time. Once again, a number of features corresponding to the 104 reflection of calcite were observed, and in this case, they were preserved after 1D data reduction [Fig. 2 ▸(*c*)]. However, the signal-to-noise ratio and background subtraction efficiency were still quite low, making automated frame selection and processing impossible. Individual frames containing reflections had to be selected by eye and processed individually, and no calcite reflections weaker than the 104 were observed.

With these promising results, we increased the flow channel width in order to obtain a beam path length that would optimize the competing X-ray scattering and attenuation of the sample. According to the equation, *I* ≃ *I*_0_exp(−*Ad*), the maximum transmitted intensity (*I*) occurs at a path length (*d*) of ∼0.98 mm, where *I*_0_ is the incident beam intensity and *A* is the material- and wavelength-dependent linear attenuation coefficient [calculated from NIST tables using water and Cu *K*α X-rays (1.541 Å)] (Ghazal *et al.*, 2016 ▸). Therefore, we adapted a millifluidic setup previously utilized at a synchrotron facility (Levenstein, Wayment *et al.*, 2020 ▸) with a 1 mm inner diameter Kapton tube for mounting on the diffractometer [Figs. 2 ▸(*a*) and S1]. Slurries of pre-made calcite or form I paracetamol (PCM I) crystals were used to form water-in-oil plugs. The concentration of crystals within the plugs was on the order of 0.4–1 wt% (see the supporting information for experimental details), which is comparable to the final mass concentration of calcite crystals in the microfluidic experiments described above (0.5 wt%). Excellent scattering statistics were obtained for both materials with 25 ms exposure times, and frames containing crystalline reflections could be processed and combined using an automated serial approach [Figs. 2 ▸(*d*) and 2 ▸(*e*)]. Based on the beam size and the plug velocities, the dwell time of a crystal in the beam was ∼5–6 ms. In the final composite diffraction patterns, the smaller ∼5–20 µm calcite crystals produced more powder-like Debye–Scherrer rings and the larger ∼50–150 µm PCM I crystals produced more single crystal-like diffraction spots (Fig. S4). All diffraction peaks could be indexed using reference data.

Although these final results obtained with the Synergy-R diffractometer were collected at an optimized path length, it is clear that the differences between the Xeuss 2.0 and the Synergy-R setups affected the overall quality of the WAXS/XRD data obtained from both instruments. Despite having comparable total source fluxes, the systems have vastly different upstream optical configurations: the Xeuss for minimizing divergence and the Synergy-R for maximizing flux. The Synergy-R is also designed for producing small beam sizes, whereas the standard beam size of the Xeuss had to be reduced to fit into the microfluidic channel. Ultimately, these differences resulted in a three order of magnitude difference in flux (ph s^−1^) and a four order of magnitude difference in flux density (ph s^−1^ µm^2^) in the beams provided by both instruments during our experiments (Table 1 ▸).

Though sub-second microfluidic SAXS analysis of silica nanoparticles was possible with the collimated optical configuration of the Xeuss, it was not possible to analyze WAXS in microfluidic flows. This suggests that a similar microfocused configuration to the Synergy-R could enable not only WAXS analysis, but also the faster collection of SAXS data at significantly lower particle concentrations on the Xeuss instrument. We recognize that the ability to resolve small angles is limited by the X-ray beam divergence and more specialized focusing optics that minimize divergence would be required for performing SAXS. Therefore, our study shows that a microfocused beam, given its design is already optimized for small single crystals, is a better option for collecting data from flowing droplets, regardless of the quality and strength of each instrument.

## 3. Conclusions

In summary, we investigated the potential of state-of-the-art laboratory X-ray instruments for conducting rapid serial scattering and diffraction studies of materials in dilute aqueous solution. Experiments with an X-ray compatible microfluidic device demonstrated that it was possible to detect diffraction during a precipitation process with an X-ray exposure time of just 25 ms and to collect sub-second SAXS patterns from nanoparticles suspended within flowing droplets. Though following ultrafast processes (≲1 ms) will likely require large-scale X-ray facilities for the foreseeable future, the performance of laboratory X-ray systems is sufficient for monitoring many dynamic samples and reactions relevant to crystal engineering, condensed matter physics, flow chemistry, and other related fields.

Both SAXS and WAXS/XRD analyses are possible as long as the beam optics and solution path length are optimized for the given material, X-ray wavelength and reaction conditions. Indeed, using a simple millifluidic device with an optimized path length and microfocused X-ray source, we successfully acquired serial powder diffraction patterns from segmented flows of organic and inorganic crystal slurries in which flows were transiting the beam in ∼5 ms. Our findings thus additionally highlight the enormous potential of millifluidic devices, which provide many of the benefits of microfluidic devices, such as lower reagent consumption and better batch-to-batch consistency than compared with bulk, but with a longer beam path through the solution. In the future, millifluidic devices comprising narrow microfluidic mixers could even be utilized to achieve ultrafast mixing and greater time resolution while still maximizing scattering intensity.

Finally, while this work was primarily motivated to study precipitation processes for materials science, it may also have major implications for structural biology. We envision a process of serial laboratory crystallography in which macromolecular powder diffraction (Shapiro *et al.*, 2008 ▸) is used for both structure refinement (Margiolaki & Wright, 2008 ▸) and mix-and-inject studies (Schmidt, 2013 ▸) to complement synchrotron and XFEL measurements, if crystals are of sufficient diffraction quality. Therefore, these findings open time-resolved serial X-ray scattering experiments to a much wider community of researchers who can now benefit from these methods without the need of a synchrotron facility.

## 4. Related literature

The following references are cited in the supporting information: Levenstein (2019 ▸); Mazutis *et al.* (2019 ▸).

## Supplementary Material

Experimental methods and supporting figures. DOI: 10.1107/S2052252522007631/lq5047sup1.pdf

## Figures and Tables

**Table 1:** X-ray source and beam characteristics

Instrument	Source	Energy (keV)	Beam size, H × W (µm)	Flux at sample (ph s^−1^)	Flux density (ph s^−1^ µm^−2^)
Xeuss 2.0 SAXS/WAXS Beamline	MetalJet D2+ (Ga)	9.24	∼250 × 250	3.7 × 10^6^	5.9 × 10^1^
XtaLAB Synergy-R Diffractometer	PhotonJet-R (Cu)	8.05	∼140 × 140	5.7 × 10^9^	2.9 × 10^5^
