# Supplementary material for: Serial small- and wide-angle X-ray scattering with laboratory sources
Source: IUCrJ. 2022 Aug 13;9(Pt 5):538–43. doi: 10.1107/S2052252522007631 (PMC9438489; doi:10.1107/S2052252522007631)
Supplement: Supplementary file 1 [file m-09-00538-sup1.pdf]

IUCrJ

Volume 9 (2022)

Supporting information for article:

Serial small- and wide-angle X-ray scattering with laboratory sources

Mark A. Levenstein, Karen Robertson, Thomas Turner, Liam Hunter, Cate O'Brien, Cedrick O'Shaughnessy, Alexander N. Kulak, Pierre Le Magueres, Jakub Wojciechowski, Oleksandr O. Mykhaylyk, Nikil Kapur and Fiona C. Meldrum

Supporting Information

Serial Small- and Wide-Angle X-ray Scattering with Laboratory Sources

Mark A. Levenstein,*[1] Karen Robertson,[2] Thomas Turner,[3] Liam Hunter,[3] Cate O'Brien,[4] Cedrick O'Shaughnessy,[3] Alexander N. Kulak,[3] Pierre Le Magueres,[5] Jakub Wojciechowski,[6] Oleksandr O. Mykhaylyk,[4] Nikil Kapur,*[7] and Fiona C. Meldrum*[3]

[1] Université Paris-Saclay, CEA, CNRS, NIMBE, 91191, Gif-sur-Yvette, FR, *Email: mark.levenstein@cea.fr*

[2] Department of Chemical and Environmental Engineering, University of Nottingham, University Park, Nottingham NG7 2RD, UK

[3] School of Chemistry, University of Leeds, Woodhouse Lane, Leeds LS2 9JT, UK, *Email: F.Meldrum@leeds.ac.uk*

[4] Soft Matter Analytical Laboratory, Department of Chemistry, The University of Sheffield, Brook Hill, Sheffield S3 7HF, UK

[5] Rigaku Americas Corporation, 9009 New Tails Drive, The Woodlands, TX 77381, US

[6] Rigaku Europe SE, Hugenottenallee 167, 63263 Neu-Isenburg, DE

[7] School of Mechanical Engineering, University of Leeds, Woodhouse Lane, Leeds LS2 9JT, UK, *Email: N.Kapur@leeds.ac.uk*

Experimental Methods:

Microfluidic device design and operation: Microfluidic experiments were performed with a previously described polymer insert-based microfluidic platform (Fig. S2a).[1] Briefly, a central polytetrafluoroethylene (PTFE) insert (300 µm thickness) bearing a serpentine microchannel design (300 µm width) was sealed by two low X-ray scattering Kapton films (75 µm thickness) and two silicone rubber gaskets (300 µm thickness). These inserts were held together by top and bottom polymethyl methacrylate (PMMA) base plates, which provided ¼-28 UNF world-to-chip connections for 1/16" OD fluorinated ethylene propylene (FEP, Cole-Parmer) tubing using standard flat-bottom, flangeless, HPLC fittings (Upchurch Scientific). Prior to experiments, channels were rendered hydrophobic by treatment with Aquapel (Pittsburgh Glass Works) using the protocol described by Maritus et al.[2] Flows were driven by NEMESYS low-pressure pumping modules (Cetoni) connected to 5 mL syringes (BD Plastipak) containing the working fluids. The approximate dwell time of particles in the beam was calculated using the equation, $t_{\mathrm{d}} = w_{beam}/v_{mean}$, where w_{beam} is the horizontal width of the X-ray beam and v_{mean} is the mean velocity of the flow. The mean velocity was calculated using the equation, $v_{mean} = Q/A$, where Q is the volumetric flow rate applied by the pumps and A is the cross-sectional area of the flow channel. After experiments, devices were disassembled and cleaned with deionised water and ethanol before reuse. In cases of crystal fouling of the channels, inserts were cleaned in 1 M HCl prior to water and ethanol rinsing.

Calcite precipitation with bioactive glass: According to the previous method,[3] aqueous solutions of 300 mM $CaCl_2$ and 300 mM Na_2CO_3 were mixed at a 3-inlet Y-junction with a buffer flow of water to produce a solution with a final equimolar concentration of 50 mM Ca^{2+} and CO_3^{2-} (2:8:2 µL min^{-1} $CaCl_2$:water:Na_2CO_3; Fig. S2b). This solution was broken into droplets at a T-junction by a perpendicular flow of FC-40 fluorinated oil (3M™ Fluorinert™) containing 2 wt% triblock copolymer surfactant at a flow rate of 20 µL min^{-1}. To induce the rapid nucleation of calcite ($CaCO_3$) crystals, the $CaCl_2$ solution was pre-loaded with 0.01 wt% of a 58S bioactive glass powder (Naomi's Nucleant, Molecular Dimensions, UK).

Millifluidic device design and operation: A millifluidic slurry delivery setup was developed for direct use on a standard Rigaku XtaLAB Synergy-R Diffractometer. Slurries of calcite or form I paracetamol (PCM I) crystals in deionised water were actively stirred using an overhead impeller to ensure continued suspension of the crystals in the source reservoirs. The calcite crystals ($CaCO_3$ powder, 98% ≤50 µm particle size) and PCM I crystals (acetaminophen, meets USP testing specifications) were obtained from Sigma Aldrich. The slurries were delivered *via* a Vapourtec UK SF-10 peristaltic pump (flow rates of 610 µL min^{-1} for PCM I and 730 µL min^{-1} for $CaCO_3$). Carrier fluid (Galden SV110 oil, perfluoropolyether) was delivered *via* NEMESYS low-pressure pumping modules connected to 50 mL plastic syringes (flow rates of 800 µL min^{-1} for PCM I and 400 µL min^{-1} for $CaCO_3$). Segmentation of the slurry flow by the carrier fluid was achieved at a polypropylene (PP) Y-piece mixer (2 mm ID) with a counter current alignment (Fig. S5). The resultant flow traversed 1/32" ID, 1/16" OD FEP tubing into the radiation-shielded enclosure, and the effluent was then collected in a waste beaker. The FEP tubing was held in place across the beam path (Fig. S2b) using optical post-mounted V-clamps (Thorlabs) attached to double suction cup grippers. Between the two optical posts, low scattering Kapton tubing (1.0 mm ID, 1.1 mm OD) was used as an analysis window and connected to the FEP tubing via ~10 mm-long sections of flexible silicone tubing (0.9 mm ID). The Kapton tubing was treated with Aquapel to maintain the hydrophobic surface properties of the FEP tubing.[4] The

solid wt% of the delivered slurries was determined experimentally by running the slurry through a SF-10 pump and measuring the mass of solids filtered and volume of filtrate over set time periods between 1 and 4 min across four replicate experiments. An average of 0.45 $\pm$ 0.15 wt% solids was delivered from these experiments. The approximate dwell time of particles in the beam was calculated as above for the microfluidic devices.

Data processing: SAXS and WAXS data obtained with the Xeuss 2.0 SAXS/WAXS beamline (Xenocs) at the SMALL lab from the microfluidic device were not processed further because low scattering statistics prevented the development of a consistent background profile for subtraction. Rather, the raw 2D WAXS frames were directly azimuthally integrated to produce the 1D scattering curves. Raw 2D SAXS frames from silica nanoparticles were serially summed into a single composite frame and then azimuthally integrated to produce the 1D scattering curve. This curve was fit using the "polydisperse spherical model with beam convolution" tool of the open-source, Python-based, SAXS analysis software, pySAXS.[5]

Microfluidics XRD data obtained with the XtaLAB Synergy Custom diffractometer (Rigaku) at Flow-Xl were processed manually. Individual 2D frames containing crystals were subject to background subtraction using the equation:

$$\mathrm{p}_{i,j,\mathrm{processed}} = \mathrm{p}_{i,j,raw} - \alpha \cdot \mathrm{p}_{i,j,\mathrm{background}} \qquad (1)$$

where $\mathrm{p}_{i,j,\mathrm{processed}}$ is each pixel (x_i, y_j) in the processed pattern, $\mathrm{p}_{i,j,\mathrm{raw}}$ is each pixel in the raw pattern, $\mathrm{p}_{i,j,\mathrm{background}}$ is each pixel in a background pattern containing the device window and solution scattering without crystals, and α is a background modifier. Here, we used $\alpha = 0.6$. After background subtraction, the pattern was smoothed if required using Weiner, median, and Gaussian image filters in MATLAB. After filtering, residual diffuse scattering was eliminated with a thresholding function:

$$\mathrm{p}_{i,j,\mathrm{processed}} = \begin{cases} 0, & \text{if } \mathrm{p}_{i,j,processed} < \mathrm{p}_{thresh} \\ \mathrm{p}_{i,j,processed}, & \text{if } \mathrm{p}_{i,j,processed} > \mathrm{p}_{thresh} \end{cases} \qquad (2)$$

where p_{thresh} is a threshold pixel intensity identified for each experiment. Here, we used $\mathrm{p}_{thresh} = 1$.

Millifluidics XRD data obtained with the XtaLAB Synergy-R diffractometer (Rigaku) at Rigaku Oxford Diffraction were processed automatically using a custom frame selection routine developed in MATLAB.[4] Briefly, frames corresponding to oil and water were distinguished using a region of interest (ROI) located over the most intense scattering band of the oil phase (Fig. S6). When the total scattering intensity of pixels in the ROI rose above a threshold value, a frame was assigned as corresponding to "oil" scattering and discarded. The remaining "water" frames were processed using the same pipeline as detailed above for the microfluidic data and then serially summed and azimuthally integrated to produce the final 1D diffraction patterns.

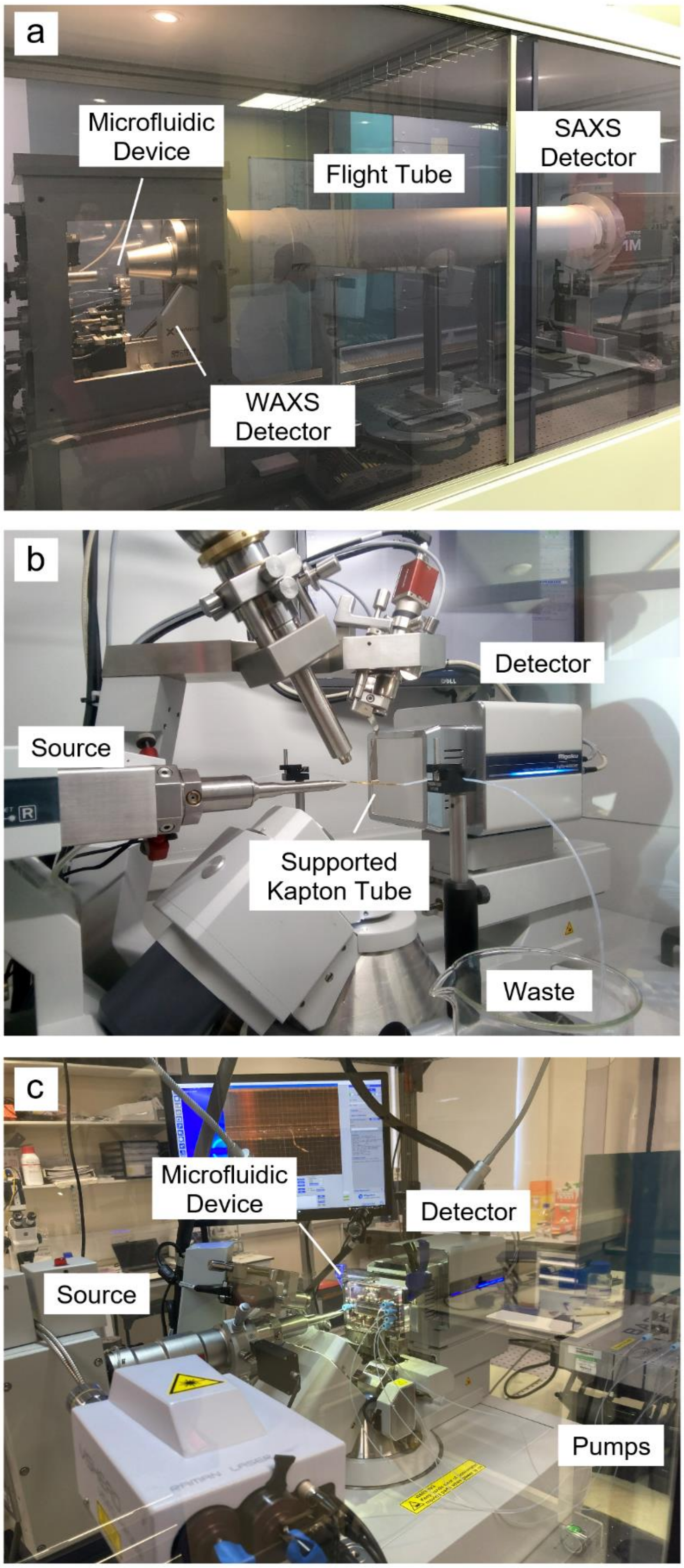

Figure S1: Experimental setups on the (a) Xeuss 2.0 SAXS/WAXS beamline at the SMALL facility, (b) the XtaLAB Synergy-R at the Rigaku Oxford Diffraction application centre and (c) the XtaLAB Synergy Custom at the Flow-Xl facility. The Synergy Custom comprises a MicroMax-007 VHF source, which has comparable characteristics to the PhotonJet-R of the Synergy-R (Table 1 in the main text).

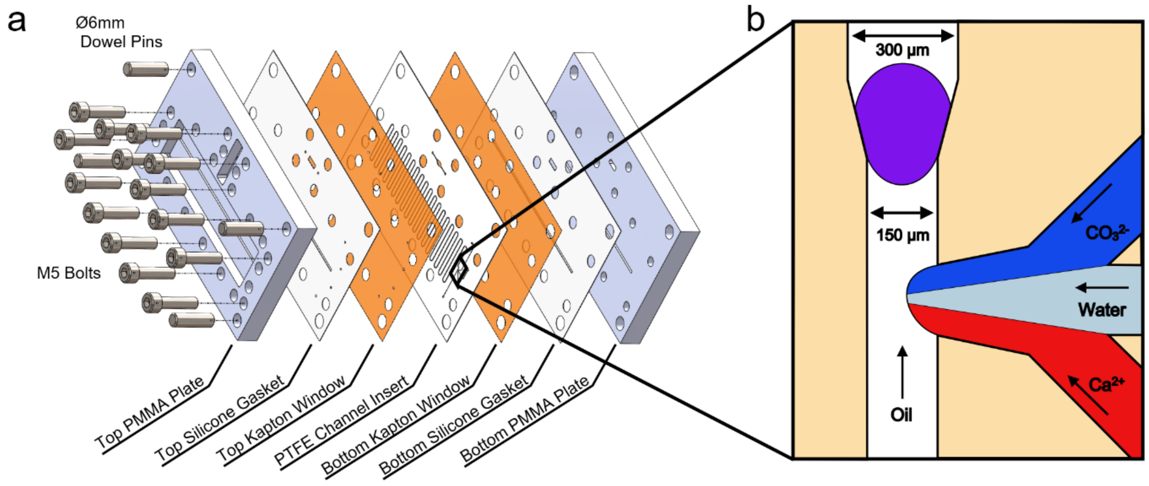

Figure S2: (a) Exploded design of the insert-based microfluidic device. (b) Illustration of the channel design and the flows utilised for the rapid calcite precipitation experiment.

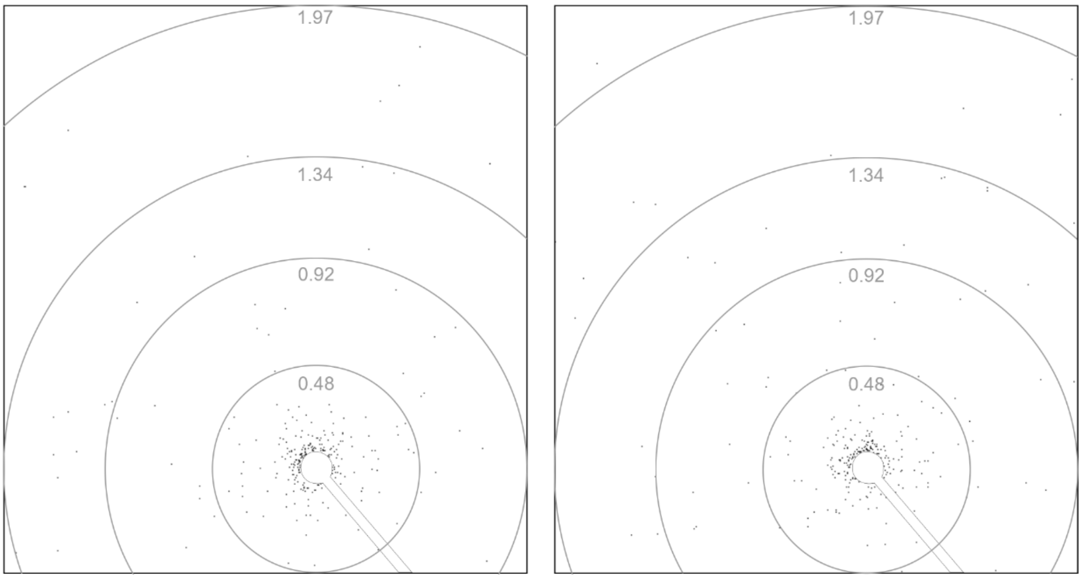

Figure S3: Two consecutive SAXS frames from the microfluidic calcite precipitation experiment corresponding to the WAXS frames shown in Fig. 1b. The scattering vector, *q*, is displayed in units of $Å^{-1}$.

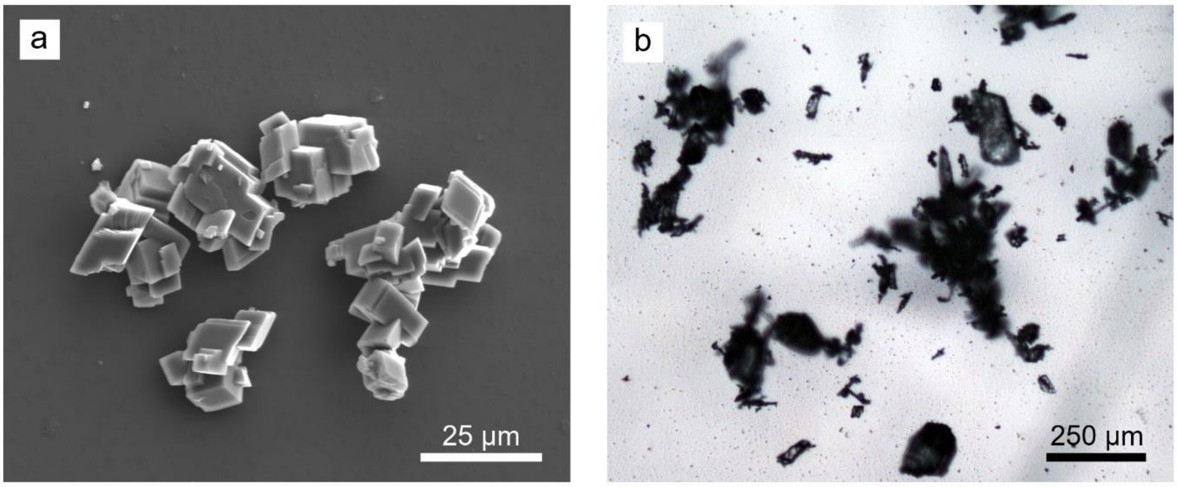

Figure S4: (a) Scanning electron micrograph of calcite powder. (b) Optical micrograph of PCM I crystals.

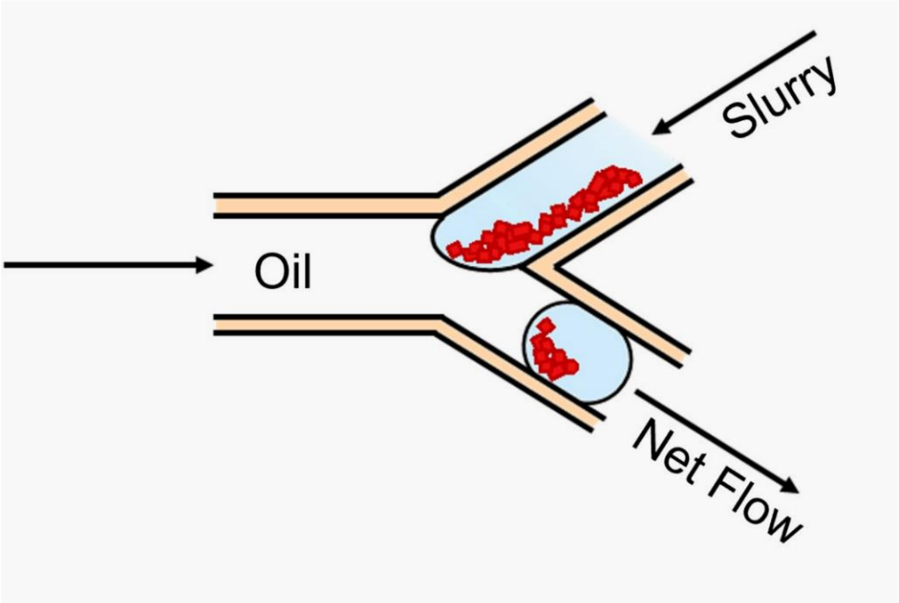

Figure S5: Illustration of the counter current alignment of the Y-mixer used for segmentation in the millifluidic slurry experiments on the XtaLAB Synergy-R diffractometer.

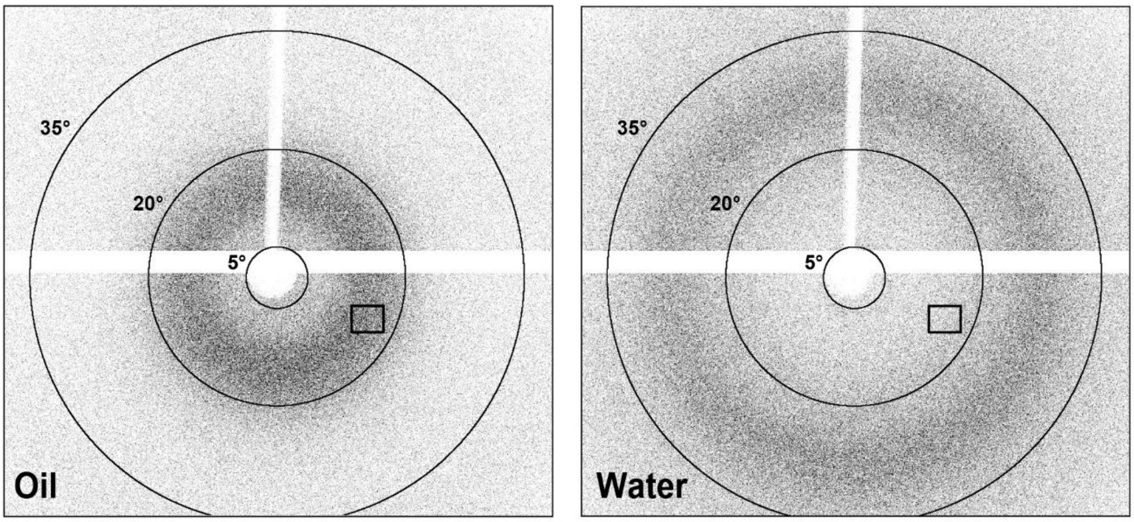

Figure S6: 25 ms exposure frames displaying the characteristic scattering of Galden® PFPE fluorinated oil and water in millifluidic experiments with the XtaLAB Synergy-R diffractometer. The black boxes show the region of interest (ROI) used to distinguish between oil and water in the automated frame selection and processing routine. The scattering angle, 2θ, is shown in degrees.

Supplementary References:

1. M. A. Levenstein, Y.-Y. Kim, L. Hunter, C. Anduix-Canto, C. González Niño, S. J. Day, S. Li, W. J. Marchant, P. A. Lee, C. C. Tang, M. Burghammer, F. C. Meldrum and N. Kapur, *Lab Chip*, 2020, **20**, 2954-2964.
2. L. Mazutis, J. Gilbert, W. L. Ung, D. A. Weitz, A. D. Griffiths and J. A. Heyman, *Nat. Protoc.*, 2013, **8**, 870-891.
3. M. A. Levenstein, C. Anduix-Canto, Y.-Y. Kim, M. A. Holden, C. González Niño, D. C. Green, S. E. Foster, A. N. Kulak, L. Govada, N. E. Chayen, S. J. Day, C. C. Tang, B. Weinhausen, M. Burghammer, N. Kapur and F. C. Meldrum, *Adv. Funct. Mater.*, 2019, **29**, 1808172.
4. M. A. Levenstein, PhD, University of Leeds, 2019.
5. O. Taché, O. Spalla, A. Thill, D. Carriere, F. Testard and D. Sen, pySAXS, an Open Source Python Package and GUI for SAXS Data Treatment, https://iramis.cea.fr/en/Phocea/Vie_des_labos/Ast/ast_sstechnique.php?id_ast=1799.
